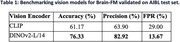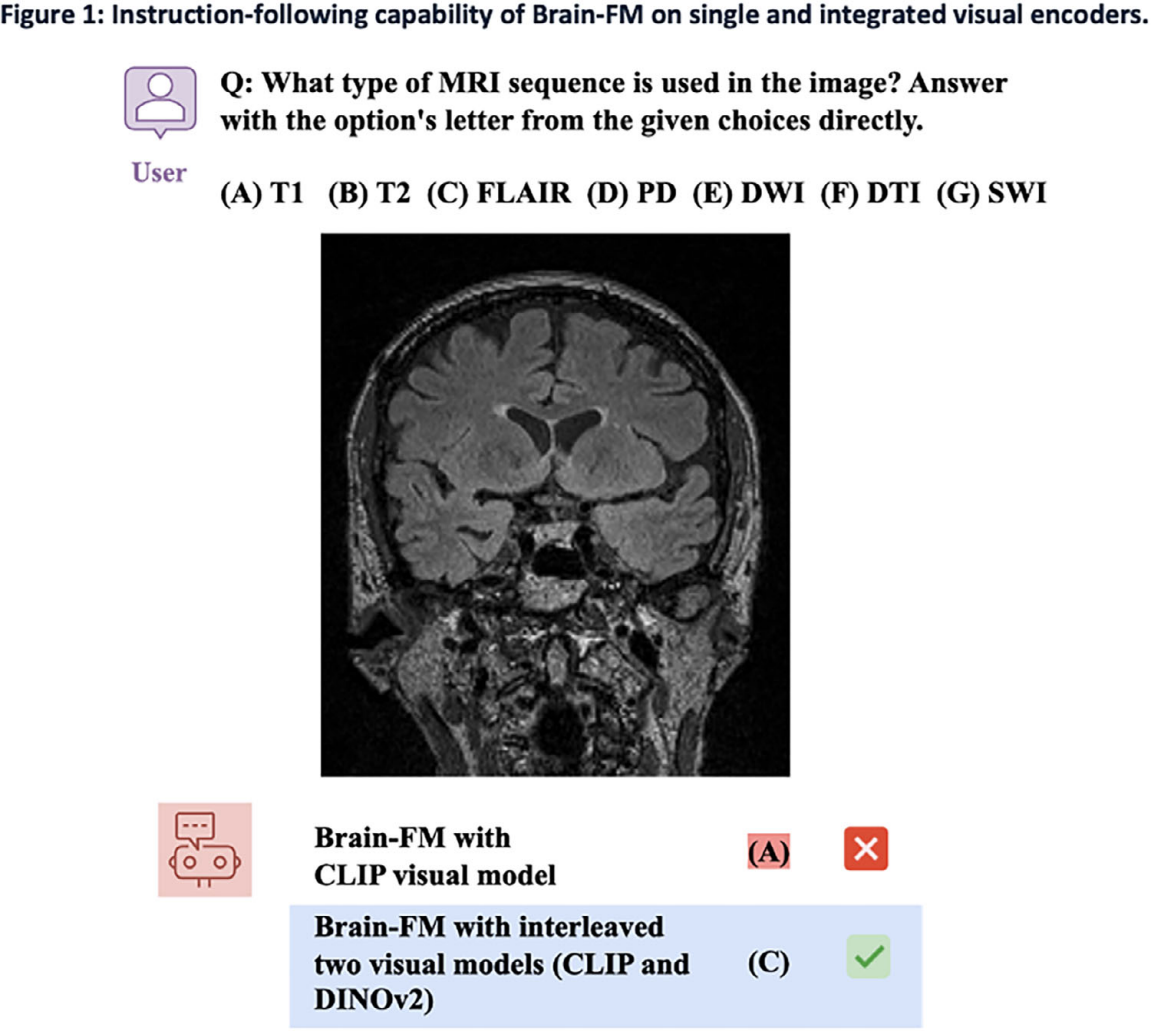# Brain‐FM: A Multimodal Foundation Model for Visual Question Answering in Brain Health Diagnostics

**DOI:** 10.1002/alz70856_104430

**Published:** 2025-12-26

**Authors:** Somayeh Ebrahimkhani, Guimeng Liu, Tianze Yu, Xuling Lin, Adeline Su Lyn Ng, Simon Kang Seng Ting, Shahul Hameed, Eng King Tan, Wing Lok Au, Kok Pin Ng, Ngai‐Man Cheung

**Affiliations:** ^1^ Singapore University of Technology and Design (SUTD), Singapore, Singapore; ^2^ National Neuroscience Institute, Singapore, Singapore

## Abstract

**Background:**

Despite advancements in AI for diagnosing neurodegenerative diseases (NDs) like Alzheimer's (AD), existing models are task‐specific and lack diagnostic reasoning, limiting their ability to interpret complex clinical and imaging data. Leveraging recent progress in multimodal foundation models (FMs) for healthcare, we developed Brain‐FM, a FM‐powered visual question answering (VQA) system. Brain‐FM enhances brain health diagnostics using image‐text data from scientific literature and is adaptable to diverse healthcare tasks and subpopulations.

**Method:**

We constructed PMC‐Dementia dataset, a curated dataset of image‐caption pairs, that were sourced from 4.5 million scientific publications, focusing on ND subtypes. To study the FM's ability in interpreting the visual inputs, we employed two visual encoders, CLIP (Radford et al., 2021) and DINOv2 (Oquab et al., 2024) and integrated their features to construct our Brain‐FM. Next, we randomly selected magnetic resonance imaging (MRI) images of 200 participants from the AIBL (Australian Imaging Biomarkers and Lifestyle Study of Ageing) dataset to generate a VQA dataset. We then evaluated the visual reasoning capabilities of our Brain‐FM in identifying the correct MRI plane, sequence (such as T1, T2‐weighted and Fluid Attenuated Inversion Recovery, FLAIR) and diagnostic reasoning.

**Result:**

We found that different visual encoders had varying visual reasoning performance when tested on our curated AIBL dataset (Table 1). The integration of DINOv2 with CLIP enhanced the Brain‐FM's visual interpretation and instruction‐following capabilities with improved accuracy and precision. Figure 1 illustrates Brain‐FM's ability to follow our instruction and answer our visual question accurately when using DINOv2 with CLIP compared to CLIP alone.

**Conclusion:**

This study introduces Brain‐FM, a multimodal foundation model tailored for brain health applications, leveraging visual‐text data to enhance diagnostic reasoning. Experimental results show that mixing the visual features achieves high accuracy when finetuned on PMC‐Dementia dataset. Brain‐FM represents a significant step toward more flexible, generalizable AI systems for brain health diagnostics and reasoning.